# NUF2 is correlated with a poor prognosis and immune infiltration in clear cell renal cell carcinoma

**DOI:** 10.1186/s12894-023-01258-x

**Published:** 2023-05-03

**Authors:** Baishu Zheng, Shunde Wang, Xiaoyu Yuan, Junyong Zhang, Zhongjie Shen, Chengguo Ge

**Affiliations:** 1 Department of Urology, The Second Affiliated Hospital of Chongqing Medical University, 76 Linjiang Road, Yuzhong district, Chongqing, 400010 China; 2 Department of Urology, Chenjiaqiao Hospital of Shapingba District, Chongqing, China

**Keywords:** NUF2, Clear cell renal cell carcinoma, Tumor immune infiltration, Immune cells

## Abstract

**Background:**

Clear cell renal cell carcinoma (ccRCC) is one of the most common malignancies. Recently, immunotherapy has been considered a promising treatment for metastatic ccRCC. NUF2 is a crucial component of the Ndc80 complex. NUF2 can stabilize microtubule attachment and is closely related to cell apoptosis and proliferation. This research is dedicated to investigating the role of NUF2 in ccRCC and the possible mechanisms.

**Methods:**

First, analysis of NUF2 mRNA expression levels in ccRCC and normal tissues by The Cancer Genome Atlas (TCGA) database and further verified by analysis of independent multiple microarray data sets in the Gene Expression Omnibus (GEO) database. Moreover, we evaluated and identified correlations between NUF2 expression, clinicopathologic variable, and overall survival (OS) in ccRCC by various methods. We investigated the relationship between NUF2 and tumor immune infiltration and the expression of corresponding immune cell markers via the Gene Expression Profiling Interactive Analysis (GEPIA) and Tumor Immune Estimation Resource (TIMER) databases. Then, we performed functional enrichment analysis of NUF2 co-expressed genes using R software and protein-protein interactions (PPIs) using the search tool used to retrieve interacting genes/proteins (STRING) databases.

**Results:**

We discovered that NUF2 mRNA expression was upregulated in ccRCC tissues and was associated with sex, grade, pathological stage, lymph node metastasis, and worse prognosis. In addition, NUF2 was positively linked to tumor immune cells in ccRCC. Moreover, NUF2 was closely related to genetic markers of different immune cells. Finally, functional enrichment and protein–protein interaction (PPI) analysis suggested that NUF2 and its closely related genes may be involved in the regulation of the cell cycle and mitosis. Our results suggested that NUF2 is correlated with a poor prognosis and immune infiltration in ccRCC.

## Introduction

Renal cell carcinoma (RCC) is one of the most common malignancies worldwide, accounting for 90% of renal tumors, of which approximately 80–90% are clear cell renal cell carcinoma (ccRCC) [1, 2]. Although early-stage or locally advanced renal cancer can be completely cured by surgical resection, local recurrence or distant metastasis still occurs in about 30% of patients within 5 years. The 5-year survival rate is less than 10% [3]. Due to the poor sensitivity of ccRCC to conventional chemoradiotherapy, immunotherapy and targeted therapy have recently been used as the first-line treatment for ccRCC metastasis, but the therapeutic effect is not satisfactory [4]. This is mainly because the mechanism of occurrence and development of ccRCC is not fully understood; therefore, there are very limited therapeutic targets meaningful in clinical practice [5]. Consequently, it is critical to deepen the exploration of the pathogenesis of ccRCC and to identify novel and meaningful parameters as diagnostic biomarkers and therapeutic targets for the treatment of ccRCC patients.

NUF2 is a crucial component of the Ndc80/NUF2 complex. As part of a linker between the kinetochore and tubulin subunits of the spindle, it can stabilize microtubule attachment and is closely related to cell apoptosis and proliferation. Disruption of NUF2 could induce defects in kinetochore attachment defects and spindle checkpoints and ultimately induce mitotic cells to undergo cell death [6–8]. NUF2 plays an important role in tumorigenesis and is upregulated in multiple human cancers, including serous adenocarcinoma, liver cancer, colorectal cancer, pancreatic cancer, ovarian cancer, lung cancer, gastric cancer and bladder cancer [9–13]. In stomach, ovarian and pancreatic cancer cell lines, gene knockout of NUF2 remarkably delays cell growth and increases cell apoptosis [9–11]. Up-regulated NUF2 has been reported to be associated with poorer prognosis in patients with hepatocellular carcinoma and non-small cell lung cancer. In addition, NUF2 can be considered a meaningful biomarker for the diagnosis of various tumors and immune therapy [10]. These findings strongly suggest a potential role for NUF2 in tumorigenesis and tumor development. However, the potential role and mechanism of NUF2 in ccRCC remain unclear.

In this study, we first performed differential expression analysis using the Gene Expression Omnibus (GEO) database, The Cancer Genome Atlas (TCGA) database and its derived online tools. Secondly, we investigated the relationship between NUF2 and clinicopathological features. Then,we analyzed the relationship between NUF2 expression and prognosis of ccRCC patients. In addition, we identified specific associations between NUF2 expression and infiltrating immune cells, immune cell markers or immune checkpoints in ccRCC. Finally, we performed functional annotation and protein interaction analysis of NUF2 and its related genes. Collectively, our study shows that NUF2 is likely to be a meaningful gene in the occurrence and development of ccRCC and an effective biomarker for the diagnosis and prognostic assessment of ccRCC.

## Methods

### TCGA and GEO database analysis

The TCGA database(https://portal.gdc.cancer.gov/cart) is a free data portal that contains information derived from a large cancer genome project, providing scholars and researchers withclinical and pathological information of 33 cancers. In our study, the main information, including NUF2 mRNA expression, clinicopathologic features and survival data of ccRCC patients, was derived from the TCGA database. Subsequent analyses were based on RNA-seq data from the TCGA database.

The GEO database(https://www.ncbi.nlm.nih.gov/geo/) is a comprehensive gene expression library in the National Center of Biotechnology Information. In this study, all of the NUF2 data representation validation sets in ccRCC were downloaded from this database, including four independent data sets (GSE53000, GSE57757, GSE46699 and GSE36895).

### UALCAN analysis

The UALCAN database(http://ualcan.path.uab.edu/index.html) is an efficient online analysis and mining cancer data tool, mainly used for the analysis of multiple cancer types in the TCGA database. It helps medical researchers with gene identification, differential expression analysis and survival analysis of genes [14]. In our study, the specific connection between NUF2 expression and clinicopathologic features related to ccRCC was analyzed based on the UALCAN database.

### GEPIA analysis

The GEPIA database (http://gepia.cancer-pku.cn/index.html) is an online tool for expression analysis and interactive analysis of cancer-related and normal genes on the basis of TCGA and Genotype-Tissue Expression (GTEX) data [15]. In our study, we analyzed the differential expression of NUF2 between ccRCC samples and normal samples and its correlation with corresponding markers for immune cells by GEPIA.

### TIMER analysis

The Tumor Immune Estimation Resource (TIMER) is a web server for analyzing the level of immune infiltration in different tumor types (http://timer.comp-genomics.org/). According to the information of more than 10,000 samples of different cancer types in the TCGA database, the immune infiltration level was analyzed by specific algorithms in the TIMER database. This database can be used to determine the level of tumor invasion according to differential gene expression [16]. This study assessed the correlation between infiltrating immune cell levels and NUF2 expression in ccRCC. The TIMER tool was also used to evaluate and verify genes significantly associated with NUF2 in the GEPIA database.

### LinkedOmics analysis and Kaplan–Meier analysis

The LinkedOmics database(http://www.linkedomics.org/) is a third-party online analysis website of TCGA data [17]. The Kaplan-Meier plotter (http://kmplot.com/ananysis/) was used for prognostic analysis. It contains more than 12,000 samples with survival data for 21 cancer types [18]. This study analyzed the relationship between NUF2 expression in ccRCC and OS with the assistance of Kaplan–Meier plotter and LinkedOmics.

### Functional annotation and PPI network analysis

We screened genes closely related to NUF2 by the R package “stat” in R (v3.6.3) (cor > 0.6, p < 0.05) and functionally annotated these significantly related genes using Gene Ontology (GO) and Kyoto Encyclopedia of Genes and Genomes (KEGG) pathway enrichment analysis [19].

In addition, The STRING database (https://string-db.org/) [20] is a website portal for searching known proteins and predicting PPIs. Using this online tool, we determined the relationships between NUF2 and interacting proteins. The correlation coefficient of the network was > 0.7, which is considered to be meaningful.

### Immunohistochemistry analysis

We performed immunohistochemical validation using tissue from patients with cCRCC in our hospital. HE staining was performed on paraffin sections. The details of these experiments have been previously described. All of the above methods are carried out in accordance with the relevant guidelines and regulations.

## Results

### NUF2 mRNA expression in ccRCC patients

**Fig. 1:**
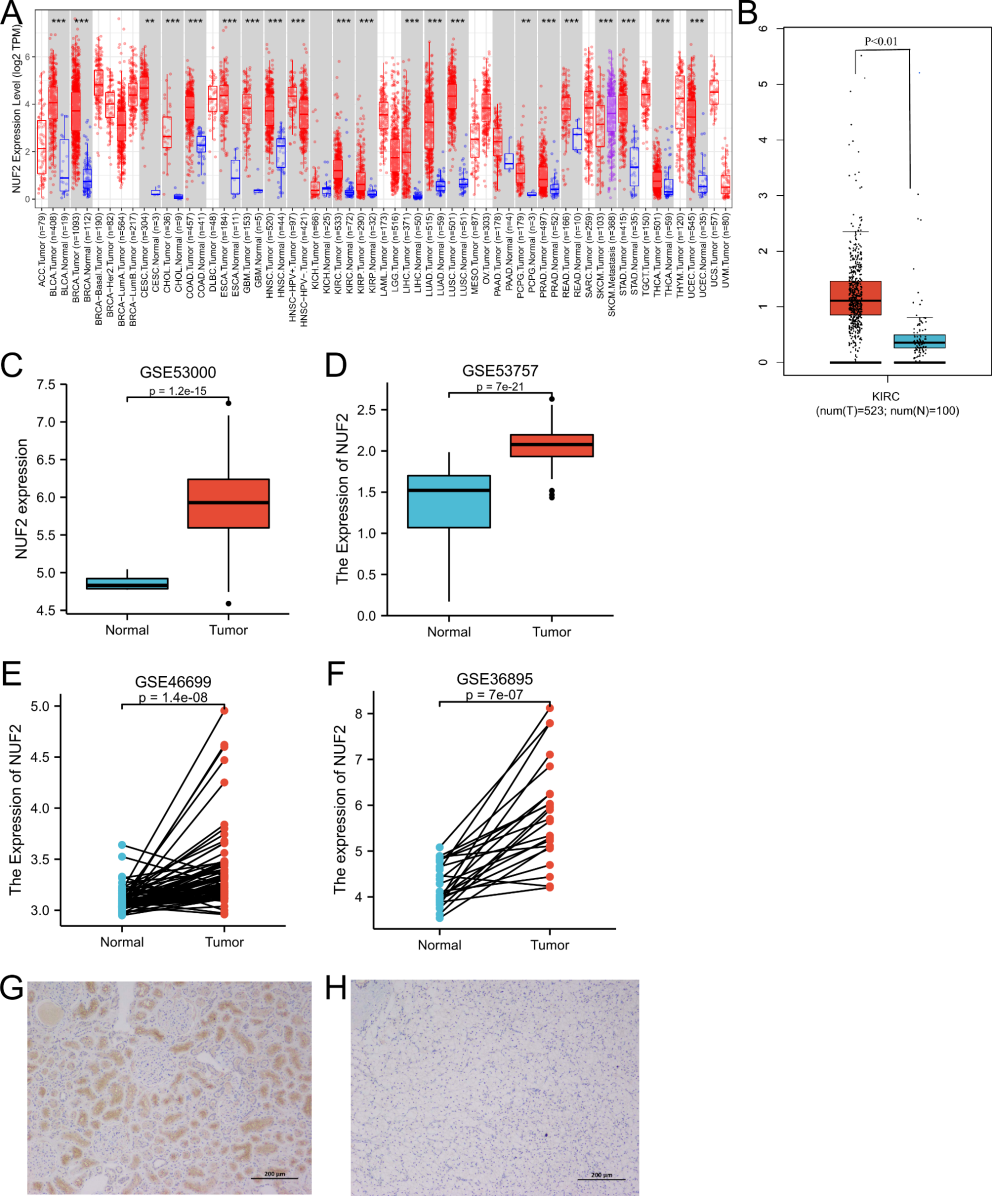
Analysis of the NUF2 gene expression in cancers. (**A**) NUF2 expression in multiple cancers (data from TCGA via TIMER) (***P < 0.001). (**B**) NUF2 mRNA expression in ccRCC tissues and normal tissues (***P < 0.001). (**C ~ D**) Validation of higher NUF2 mRNA expression in ccRCC than that in normal tissues in GSE53000 and GSE53727dataset. (**E ~ F**) Validation of higher NUF2 expression in ccRCC than that adjacent tissues in GSE46699 and GSE36895 dataset.(G-H)NUF2 protein levels in normal kidney sample(right) and ccRCC sample(left)

Evaluation of NUF2 transcript levels differentially expressed in multiple cancer samples via the TIMER database. NUF2 transcription levels were found to be high in a variety of human tumor samples, including ccRCC and 15 other kinds of cancers, such as hepatocellular carcinoma, Esophageal squamous cell carcinoma, gastric adenocarcinoma, urothelial carcinoma, lung cancer, glioma, ovarian cancer, breast cancer, cervical squamous cell carcinoma, colorectal cancer, pancreatic cancer and prostate adenocarcinoma (Fig. 1A). Furthermore, the transcript levels of NUF2 in ccRCC samples and normal samples were compared via the GEPIA database (Fig. 1B). The results suggested that the transcript level of NUF2 in ccRCC patients was notably upregulated. We retrieved four microarray data sets from the GEO database (GSE53000 GSE53757, GSE46699, GSE36895) and assessed the expression of NUF2 in ccRCC and normal samples to further validate the authenticity and accuracy of the results of NUF2 expression in ccRCC (Fig. 1C-F). In addition, immunohistochemistry confirmed high expression of NUF2 in ccRCC specimens (Fig. 1G-H). Our findings are in agreement with the results from these database analyses. All sets of figures showed high expression of NUF2 in ccRCC, indicating that NUF2 may play an irreplaceable role in tumor growth and development of various cancers, especially ccRCC, and may be a meaningful gene in ccRCC and other cancers.

### Correlation between clinicopathologic features and NUF2 expression in patients with ccRCC

We investigated the clinicopathological features of ccRCC patients with different NUF2 expression levels (Table 1). Patients in the high NUF2 expression group had higher clinical tumor-node-metastasis (TNM) stage, worse pathological stage and higher tumor grades than those in the low NUF2 expression group; however, there were no significant differences between the tumor group and the normal group in terms of age, gender, or race.

**Table 1:** Clinicopathological characteristics of ccRCC patients with differential NUF2 expression

Characteristic	Low expression of NUF2(n = 265)	High expression of NUF2(n = 265)	p
Age, n (%)			0.259
<=60	125 (23.6%)	139 (26.2%)	
> 60	140 (26.4%)	126 (23.8%)	
Gender, n (%)			0.084
Female	103 (19.4%)	83 (15.7%)	
Male	162 (30.6%)	182 (34.3%)	
Race, n (%)			0.284
Asian	3 (0.6%)	5 (1%)	
Black or African American	33 (6.3%)	23 (4.4%)	
White	225 (43%)	234 (44.7%)	
T stage, n (%)			< 0.001
T1	155 (29.2%)	116 (21.9%)	
T2	35 (6.6%)	34 (6.4%)	
T3	74 (14%)	105 (19.8%)	
T4	1 (0.2%)	10 (1.9%)	
N stage, n (%)			0.032
N0	119 (46.7%)	120 (47.1%)	
N1	3 (1.2%)	13 (5.1%)	
M stage, n (%)			< 0.001
M0	225 (45.2%)	195 (39.2%)	
M1	23 (4.6%)	55 (11%)	
Pathologic stage, n (%)			< 0.001
Stage I	151 (28.7%)	114 (21.6%)	
Stage II	31 (5.9%)	26 (4.9%)	
Stage III	58 (11%)	65 (12.3%)	
Stage IV	25 (4.7%)	57 (10.8%)	
Histologic grade, n (%)			0.042
G1	10 (1.9%)	4 (0.8%)	
G2	124 (23.8%)	103 (19.7%)	
G3	97 (18.6%)	109 (20.9%)	
G4	30 (5.7%)	45 (8.6%)	

**Fig. 2:**
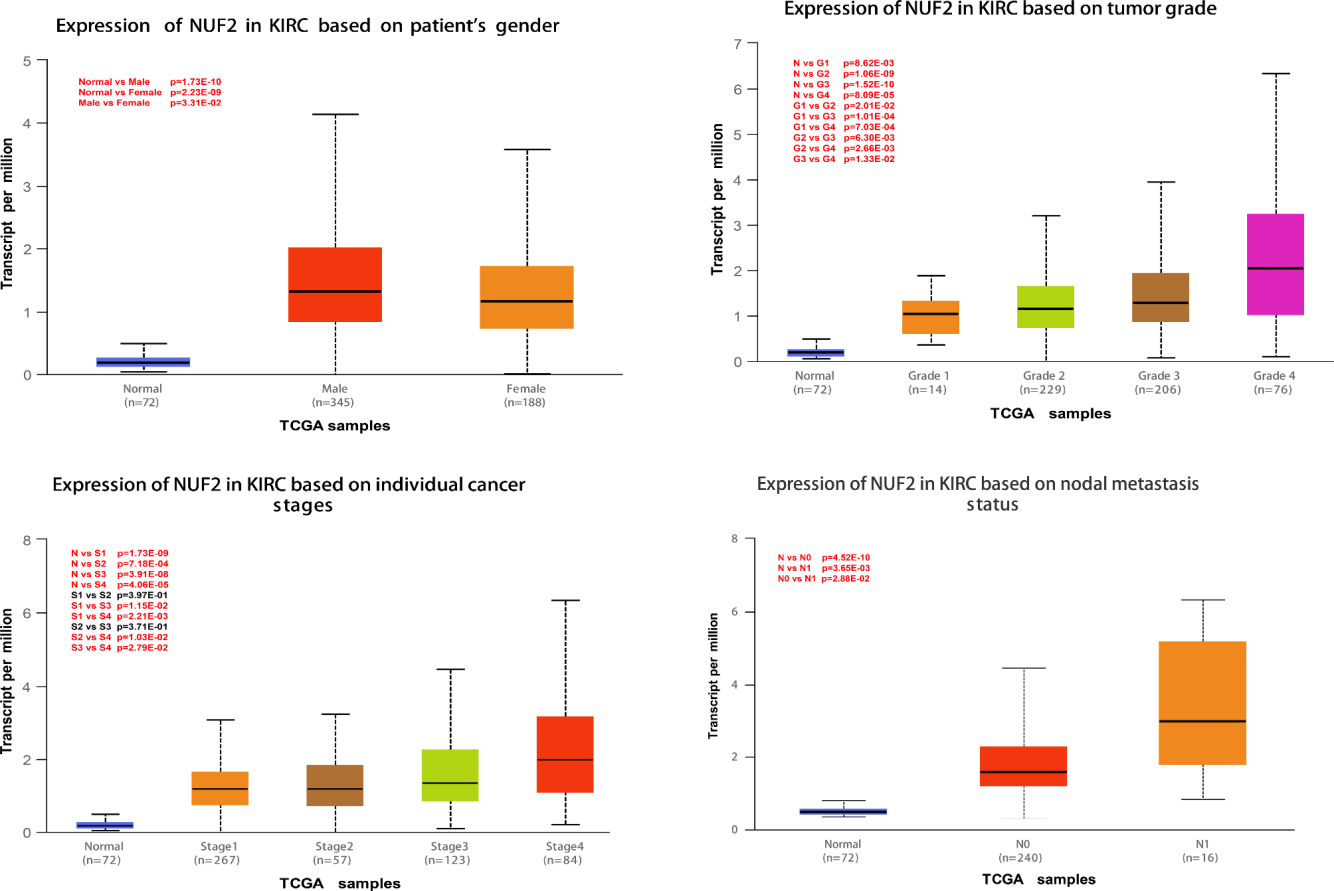
Association with NUF2 mRNA expression and clinicopathologic characteristics. (**A**) Gender(P = 0.03). (**B**) Grade(P = 8.09E-05). (**C**) Tumor stage(P = 4.05E-05). (**D**) lymph node metastasis(P = 3.65E-03).

In addition, we analyzed the expression of NUF2 in patients with different clinical characteristics through the UALCAN database. Up-regulated NUF2 in ccRCC patients was significantly correlated with gender, pathological stage, tumor grade, and lymph node metastasis, especially in men, patients with high tumor stage and grade, and multiple lymph node metastases, suggesting that high expression of NUF2 may be an adverse prognostic factor in RCC patients (Fig. 2).

### Prognostic value of NUF2 in ccRCC

**Fig. 3:**
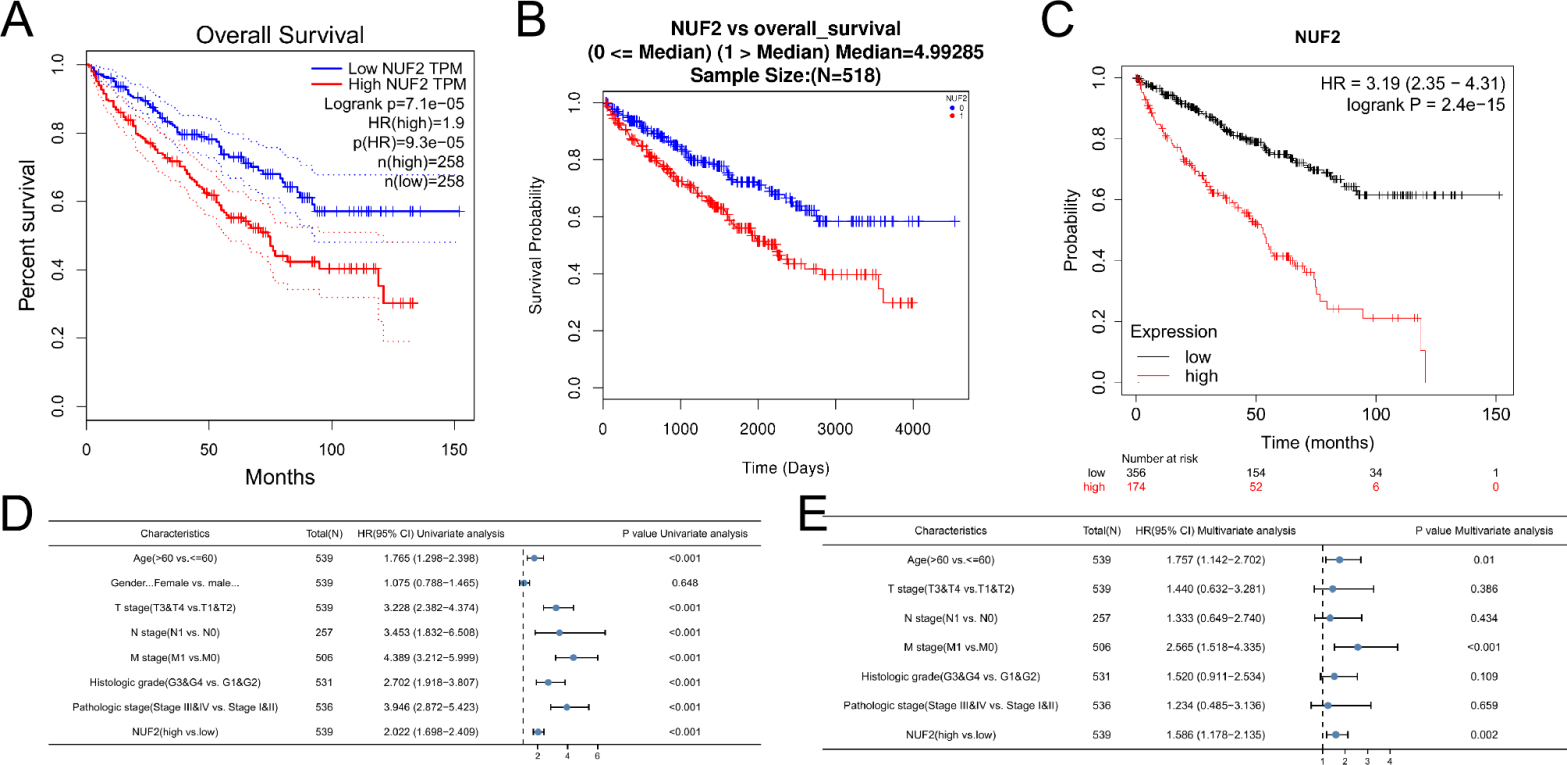
Overall survival analysis with NUF2 mRNA expression in different databases. (**A**) NUF2 expression and overall survival in ccRCC patients via GEPIA(P = 7.1E-05). (**B**) NUF2 expression and overall survival in ccRCC patients via LinkedOmics database (P < 0.01). (**C**) NUF2 expression and overall survival in ccRCC patients via Kaplan-Meier plotter analysis(P = 2.4E-15). (**D**) Uni-Cox analysis of overall survival of ccRCC patients. (**E**) Multi-Cox analysis of the overall survival of ccRCC patients

We evaluated the expression of NUF2 and verified whether it is related to the OS of ccRCC patients via GEPIA, LinkedOmics database analysis and Kaplan–Meier plotter analysis. GEPIA (Fig. 3A, P = 7.1E − 05), LinkedOmics analysis (Fig. 3B, P < 0.01) and Kaplan–Meier plotter analysis (Fig. 3C, P = 2.4e − 15) showed that high NUF2 expression was linked to poor OS in ccRCC, indicating that NUF2 is an independent prognostic factor and may be meaningful biomarker in ccRCC patients.

In addition, we performed univariate and multivariate Cox regression analyses to further assess the predictive value of NUF2 in several clinicopathological subgroups and presented the results as forest plots. As shown in Fig. 3D, gender, TNM stage, histological grade and pathological stage, and high NUF2 expression were independent risk factors for OS in the univariate Cox regression analysis. Furthermore. multifactor Cox regression analysis of the above factors (as shown in Fig. 3E), gender, M stage, and NUF2 expression still showed predictive advantages for clinical outcomes.

### Correlation between NUF2 expression level and immune cell in ccRCC

**Fig. 4:**
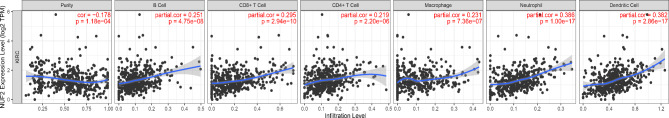
Correlation between NUF2 expression and immune cells in ccRCC tissues (n = 533)

Infiltrating immune cells may influence the development of multiple cancers. In this research, we discussed the connection between NUF2 expression and immune cells using the TIMER database. In general, a high transcript level of NUF2 was closely related to immune cell infiltration in ccRCC (Fig. 4). In ccRCC, NUF2 expression was significantly positively correlated with dendritic cell (DCs) (r = 0.382, P = 2.86E-17), B cells (r = 0.251, P = 4.75E-08), macrophages (r = 0.231, P = 7.36E-07), neutrophils (r = 0.386, P = 1.00E-17), CD4 + T cells (R = 0.219, P = 2.20E-06) and CD8 + T cells (r = 0.25, P = 5.515E-08).

### Correlations between the expression levels of NUF2 and markers of immune cells in ccRCC

The relationship between NUF2 expression and the state of tumor-infiltrating immune cells was further investigated on the basis of the expression level of immune markers in ccRCC. Immune cells in ccRCC include B cells, monocytes, NKcells, CD4 + T cells, CD8 + T cells, neutrophils, Macrophages and DCs. Furthermore, regulatory T cells (Tregs) and different T helper cells were deeply investigated.

To further analyze the role of NUF2 in tumor immunity, the GEPIA and TIMER databases were used to assess the association between NUF2 expression and immune cell markers in ccRCC tissues. We discovered that NUF2 expression had a significant positive correlation with most specific immune cell markers (Table 2), including CD8 + T cell markers (CD8A, CD8B), monocyte markers (CD86, CD115), T cell (general) markers (CD2, CD3E, CD3D),DC markers (CD11c), tumor-associated macrophage (TAM) markers (IL-10), M2 macrophage markers (CD163, VSIG4, MS4A4A), Th17 markers (STAT3), NKcell markers (KIR2DL4), Th1 markers (STAT1, STAT4, T-BET, IFN-γ), TFH markers (IL21),Th2 markers (GATA3, STAT5A), Tregs markers (TGFB1, STAT5B, CCR8, FOXP3) and T cell depletion markers (CTLA4, PD-1, TIGIT, TOX, LAG3).

**Table 2:** Correlation analysis between Nuf2 and biomarker genes of immune cells in ccRCC

Cell type	Gene markers	GEPIA		Timer	
		Cor	P-Value	Cor	P-Value
CD8 + T cell	CD8A	0.21	**9.2E − 07**	0.381	**7.90E-20**
	CD8B	0.16	**3.7E-04**	0.327	**9.18E-15**
T cell (general)	CD3D	0.18	**5.5E − 05**	0.373	**4.98E-19**
	CD3E	0.18	**2.8E − 05**	0.363	**4.60E-18**
	CD2	0.19	**8.9E − 06**	0.414	**1.97E-23**
B cell	CD19	0.068	1.2E-01	0.269	**2.78E-10**
	CD79A	0.025	5.7E-01	0.198	**4.09E-06**
Monocyte	CD86	0.17	**7.2E-05**	0.365	**3.00E-18**
	CD115	0.11	**9.4E-03**	0.278	**6.68E-11**
TAM	CCL2	-0.08	6.8E-02	0.018	6.75E-01
	CD68	0.038	3.8E-01	0.272	**1.78E-10**
	IL10	0.095	**2.9E-02**	0.319	**5.91E-15**
M1 Macrophage	NOS2	−0.033	4.5E-01	0.041	3.48E-01
	IRF5	0.072	1.0E-01	0.311	**1.89E-13**
	PTGS2	0.02	6.4E-01	0.116	**7.38E-03**
M2 Macrophage	CD163	0.22	**6.5E − 07**	0.253	**3.12E-09**
	VSIG4	0.24	**2.4E − 08**	0.294	**4.58E-12**
	MS4A4A	0.16	**3.7E-04**	0.27	**2.42E-10**
Neutrophils	CD66b	0.017	7.0E-01	0.025	5.71E-01
	CD11b	0.013	7.7E-01	0.125	4.02E + 00
	CCR7	0.024	5.8E-01	0.254	**2.65E-09**
Natural killer cell	KIR2DL1	-0.069	1.1E-01	0.026	5.46E-01
	KIR2DL3	-0.034	4.4E-01	0.062	1.50E-01
	KIR2DL4	0.13	**2.7E-03**	0.188	**1.19E-05**
	KIR3DL2	-0.05	2.6E-01	0.067	1.24E-01
	KIR3DL3	0.022	6.2E-01	0.041	3.14E-01
	KIR2DS4	-0.059	1.8E-01	-0.034	4.37E-01
Dendritic cell	HLA-DPB1	0.035	4.3E-01	0.251	**4.19E-09**
	HLA-DQB1	0.035	4.3E-01	0.131	**2.40E-03**
	HLA-DRA	0.066	1.3E-01	0.288	**1.14E-11**
	HLA-DPA1	0.04	3.6E-01	0.287	**1.55E-11**
	CD11c	0.12	**5.5E-03**	0.331	**4.25E-15**
Th1	T-bet	0.1	**2.2E-02**	0.262	**8.43E-10**
	STAT4	0.11	**9.3E-03**	0.404	**2.68E-22**
	STAT1	0.28	**9.5E − 11**	0.427	**3.30E-26**
	IFN-γ	0.4	0.0E + 00	0.465	**6.87E-30**
	TNF-a	0.044	3.2E-01	0.234	**4.81E-08**
Th2	GATA3	0.2	**2.8E − 06**	0.139	**1.26E-03**
	STAT6	-0.046	3.0E-01	0.112	**9.84E-03**
	STAT5A	0.086	**4.8E-02**	0.241	**1.73E-08**
	IL13	-0.029	5.0E-01	-0.014	7.53E-01
Tfh	BCL6	0.021	6.4E-01	0.13	**2.62E-03**
	IL21	0.24	**1.9E − 08**	0.205	**1.74E-06**
Th17	STAT3	0.025	5.7E-01	0.121	**5.31E-03**
	IL17A	0.0055	9.0E-01	0.029	5.09E-01
Treg	FOXP3	0.18	**2.3E − 05**	0.398	**1.11E-21**
	CCR8	0.14	**1.2E-03**	0.384	**3.80E-20**
	STAT5B	-0.12	**6.5E-03**	-0.062	1.54E-01
	TGFB1	0.22	**5.5E − 07**	0.227	**1.15E-07**
T cell exhaustion	PD-1	0.27	**2.2E-10**	0.358	**1.53E-17**
	CTLA4	0.33	**2.3E − 14**	0.419	**3.94E-24**
	LAG3	0.3	**1.5E − 12**	0.44	**1.32E-26**
	TIM-3	-0.037	4.0E-01	0.134	**1.99E-03**
	GZMB	0.41	**0.0E + 00**	0.293	**5.60E-12**
	TOX	0.19	**1.4E − 05**	0.446	**4.16E-21**
	TIGIT	0.3	**2.1E − 12**	0.446	**2.24E-27**

ccRCC, clear cell Renal cell carcinoma; TAM, tumor-associated macrophage; Th,T helper cell; Tfh, Follicular helper T cell; Treg, regulatory T cell; Cor, R value of Spearman’s correlation.Bold values indicate P-value < 0.05.

### Functional annotation and PPI network analysis

**Fig. 5:**
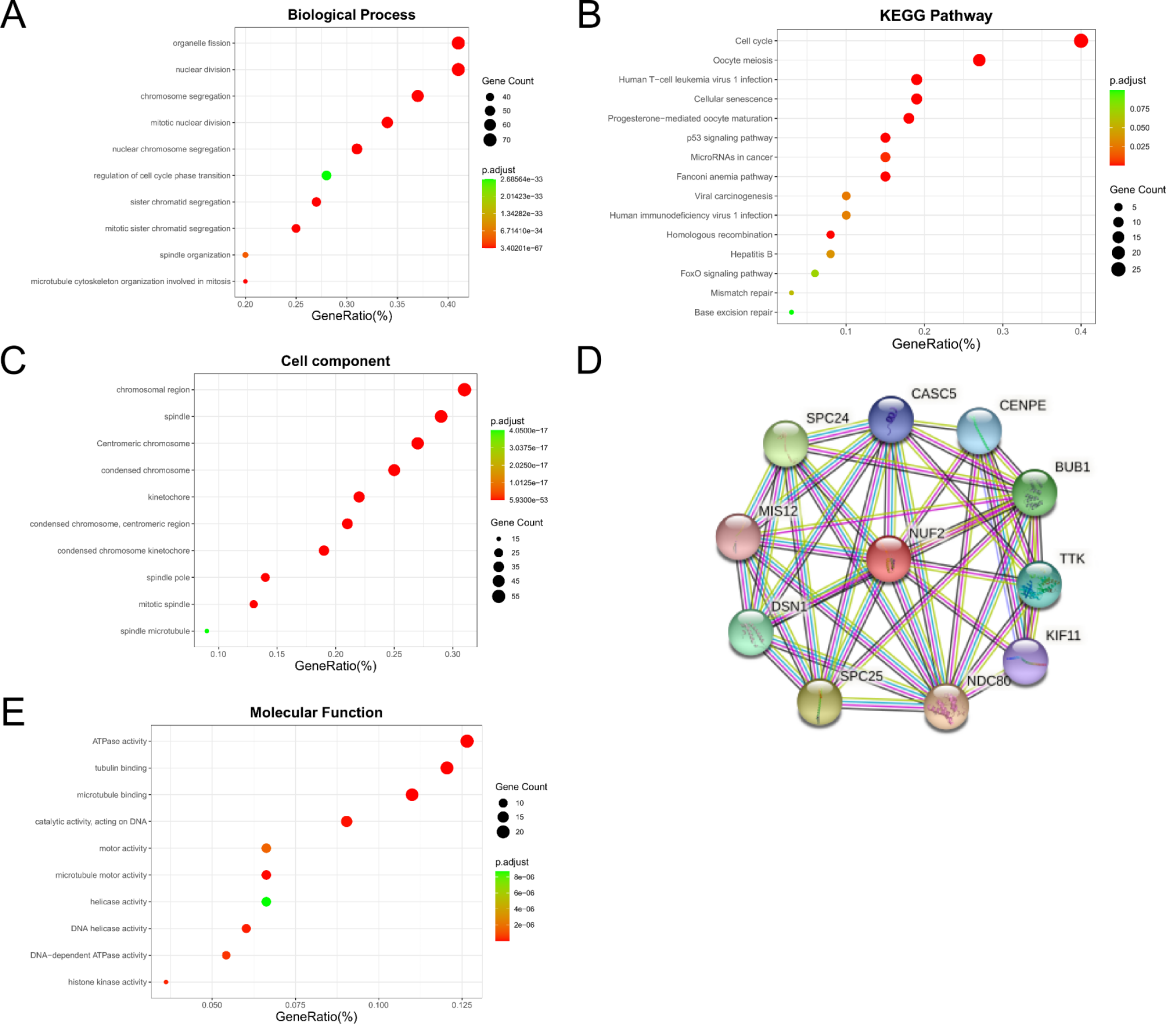
Functional enrichment and PPI. (**A**, **C**, **E**) GO terms of NUF2 in ccRCC. (**B**) KEGG pathways of NUF2 in ccRCC. (**C**) PPI network of NUF2 in ccRCC.

As shown in Fig. 5, functional enrichment analysis revealed that NUF2-related genes were mainly enriched in organelle division and chromosome segregation in biological processes and in ATPase activity and chromosomal regions in molecular functions and cellular components (Fig. 5A, C, E). According to KEGG pathway analysis, NUF2-associated genes are mainly involved in cell cycle regulation and may also be involved in classical pathways such as the P53 signaling pathway and the regulation of microRNAs in tumors (Fig. 5B).

It is well known that a more comprehensive understanding of protein functions can be achieved by studying protein interactions. We constructed an 11-point, 42-edge interactions network map of NUF2-related proteins via the STRING database (Fig. 5D), and the top 10 proteins and their genes in terms of the degree of association are as follows: NDC80, SPC25, SPC24, BUB1, DSN1, TTK, CENPE, CASC5, KIF11, and MIS12.

SPC25 and SPC24 are all part of the NDC80 complex, mainly involved in cellular chromosome segregation and spindle checkpoint activity. The 10 most correlated proteins are mainly involved in the cell cycle and essential for normal chromosome alignment, segregation and kinetochore formation during mitosis. In addition, up-regulated BUB1 is associated with a poor prognosis in ccRCC patients.

## Discussion

ccRCC, whose pathogenesis mechanism has been unclear thus far, is extremely resistant to chemoradiotherapy [1]. An in-depth study of the molecular mechanisms of ccRCC may provide important clues for the development of effective therapeutic targets or the search for promising biomarkers. ccRCC lacks specific diagnostic markers. Molecular biology methods can detect kidney cancer-related markers in urine or blood, such as DNA microsatellite alterations [21], VHL gene mutations or hypermethylation, renal cell carcinoma-specific proteins such as CA-9 expression, and upregulation of angiogenic factors such as VEGF expression [22]. However, the specificity and sensitivity of the methods are limited. Growing evidence suggests that NUF2 plays a critical role in the tumorigenesis of human cancers. But the role of NUF2 in kidney cancer is poorly understood and further research is needed.

It has been reported that NUF2 expression is significantly upregulated in many other cancers. To further clarify the function of NUF2 in ccRCC, multiple databases were analyzed and NUF2 was upregulated in ccRCC tissue compared with normal kidney tissue was found, suggesting that NUF2 is likely to be independently involved in the occurrence and development of ccRCC.

NUF2 has been previously reported to play an essential role in tumorigenesis and is upregulated in a variety of human cancers [6, 7]. The downregulation of NUF2, which regulates lncRNA AF339813, can inhibit the growth of pancreatic cancer. Moreover, the proliferation and invasion of liver cancer cells can be inhibited by targeted knockdown of NUF2, indicating that the up-regulated NUF2 is closely related to a poor prognosis in tumors [10, 11, 13]. It is basically consistent with our research results. Analysis shows that up-regulated NUF2 is strongly associated with multiple lymph node metastases, clinical stage, and histological grade. In addition, through survival analysis and verification analysis via multiple databases, we discovered that up-regulated NUF2 was obviously associated with the poor OS of ccRCC patients, strongly implying that NUF2 can be regarded as a meaningful prognostic biomarker in ccRCC.

Immunotherapy for cancer has made tremendous progress in recent years. Attention is being paid to the role of the immune response in the tumor microenvironment [23–25]. ccRCC is a tumor with a high degree of immune infiltration. NUF2 was previously reported to be closely linked to the tumor microenvironment. PD-1/PD-L1 antibodies have been used in the primary treatment of metastatic kidney cancer [26–28]. However, despite the convincing efficacy, some patients are still insensitive to PD-1 antibody treatment [28]. Recent studies have shown that the activation status of tumor-infiltrating lymphocytes and T cells is a major factor in determining the prognosis of ccRCC [29–31]. Macrophages have diverse functions and have functional plasticity mediated by microenvironment signals. M1 macrophages, known as classical macrophages, are macrophages that can produce proinflammatory cytokines and have strong abilities to kill microorganisms, but these features also easily cause tissue destruction. M2 macrophages are also called alternative activated macrophages. Some stimulating factors, such as cytokines and fungal and worm infections, are conducive to the differentiation of M2 subsets. M2 macrophages play a critical role in the response to parasites, tissue remodeling, angiogenesis and allergic diseases. [32–34]. We found a weak correlation between NUF2 upregulation and M1/M2 macrophage markers PTGS2, IRF5, CD163, VSIG4 and MS4A4A, suggesting that NUF2 plays a regulatory role in the polarization of TAMs. In addition, we discovered that the expression of NUF2 in ccRCC was positively correlated with the infiltration of various T helper cells (Th1, Th2, Tfh, and Th17) and that NUF2 regulates the T cell response. Treg cells are the main manipulators of immune suppression from the tumor microenvironment, and DCs promote tumor recurrence and metastasis by upregulating the cytotoxicity of Treg cells and downregulating the cytotoxicity of CD8 + T cells. Treg cell infiltration in tumors is associated with high pathological stage and poor prognosis in ccRCC [35–37]. We found that NUF2 mRNA expression was significantly correlated with markers of Treg cells and T cell exhaustion [38], suggesting that NUF2 may promote the Treg response, inhibit T cell-mediated immunity and improve the efficacy of immunotherapy for ccRCC. These results highlight the potential of NUF2 in regulating the recruitment and activation of infiltrating immune cells in ccRCC. Our findings offer a guide for future research to some extent. Nevertheless, NUF2 is associated with most immune cell genes, suggesting that its interactions are specific and selective, and further study is required.

It has been reported that the targeted regulation of NUF2 can inhibit cell proliferation and invasion [10–12]. In our study, based on the annotation of NUF2-related genes, cell cycle and ATPase activity were closely associated with NUF2 expression, and in addition, NUF2 was significantly enriched in the P53 signaling pathway and microRNA pathways, suggesting that NUF2 may play a key role in the occurrence of ccRCC by regulating the cell cycle or participating in the P53 signaling pathway.

In conclusion, by mining public databases, we preliminarily elucidated the relationships between the high expression of NUF2 and a poor prognosis and clinicopathologic features in ccRCC. Moreover, our current findings suggest that NUF2 may act as an oncogene by regulating the level of tumor-infiltrating immune cells and the expression of immune checkpoints. In addition, genes closely related to NUF2 are particularly abundant in the cell cycle as well as in the P53 signaling pathway. However, there are some limitations to our research. Firstly, we mined a variety of public databases, but our results should be verified with basic experiments and clinical studies. Secondly, we only focused on the transcription level, and we should analyze the mechanism of NUF2 and the upstream and downstream regulatory mechanisms at many different levels.

## Data Availability

The material supporting the conclusion of this review has been included within the article.
